# Identification of Potential lncRNAs and miRNAs as Diagnostic Biomarkers for Papillary Thyroid Carcinoma Based on Machine Learning

**DOI:** 10.1155/2021/3984463

**Published:** 2021-07-21

**Authors:** Fei Yang, Jie Zhang, Baokun Li, Zhijun Zhao, Yan Liu, Zhen Zhao, Shanghua Jing, Guiying Wang

**Affiliations:** ^1^Department of Otolaryngology-Head and Neck Surgery, The Fourth Hospital of Hebei Medical University, Hebei, China; ^2^General Surgical Department, The Fourth Hospital of Hebei Medical University, Hebei, China; ^3^General Surgical Department, The Third Hospital of Hebei Medical University, Hebei, China

## Abstract

**Background:**

Papillary thyroid carcinoma (PTC) accounts for most of the proportion of thyroid cancer (TC). The objective of this study was to identify diagnostic, differentially expressed long noncoding RNAs (lncRNAs) and microRNAs (miRNAs), contributing to understanding the epigenetics mechanism of PTC.

**Methods:**

The data of lncRNA, miRNA, and mRNA were downloaded from the Cancer Genome Atlas (TCGA) dataset, followed by functional analysis of differentially expressed mRNAs. Optimal diagnostic lncRNA and miRNA biomarkers were identified via random forest. The regulatory network between optimal diagnostic lncRNA and mRNAs and optimal diagnostic miRNA and mRNAs was identified, followed by the construction of ceRNA network of lncRNA-mRNA-miRNA. Expression validation and diagnostic analysis of lncRNAs, miRNAs, and mRNAs were performed. Overexpression of ADD3-AS1 was performed in PTC-UC3 cell lines, and cell proliferation and invasion assay were used for investigating the role of ADD3-AS1 in PTC.

**Results:**

A total of 107 differentially expressed lncRNAs, 81 differentially expressed miRNAs, and 515 differentially expressed mRNAs were identified. 11 lncRNAs and 6 miRNAs were regarded as the optimal diagnostic biomarkers for PTC. The epigenetic modifications via the above diagnostic lncRNAs and miRNAs were identified, including MIR181A2HG-FOXP2-hsa-miR-146b-3p, BLACAT1/ST7-AS1-RPS6KA5-hsa-miR-34a-5p, LBX2-AS1/MIR100HG-CDHR3-hsa-miR-34a-5p, ADD3-AS1-PTPRE-hsa-miR-9-5p, ADD3-AS1-TGFBR1-hsa-miR-214-3p, LINC00506-MMRN1-hsa-miR-4709-3p, and LOC339059-STK32A-hsa-miR-199b-5p. In the functional analysis, MMRN1 and TGFBR1 were involved in cell adhesion and endothelial cell migration, respectively. Overexpression of ADD3-AS1 inhibited cell growth and invasion in PTC cell lines.

**Conclusion:**

The identified lncRNAs/miRNAs/mRNA were differentially expressed between normal and cancerous tissues. In addition, identified altered lncRNAs and miRNAs may be potential diagnostic biomarkers for PTC. Additionally, epigenetic modifications via the above lncRNAs and miRNAs may be involved in tumorigenesis of PTC.

## 1. Introduction

Thyroid cancer (TC) accounts for 94% of the endocrine system cancers and 66% of the deaths. Clinically, TC is divided into four histological subtypes: follicular TC, anaplastic TC, medullary TC, and papillary thyroid carcinoma (PTC). Mortality of TC is low. However, the incidence and recurrence rate of PTC are increasing worldwide, especially among women [1]. Some factors appear to increase the risk of PTC, including obesity, iodine intake, iodine deficiency, environmental pollutants, exposure to ionizing radiation, noncancerous benign thyroid conditions, and history of thyroid disease (goiter, thyroiditis, or adenoma) [2–7]. Generally, PTC develops slowly and can be cured by thyroidectomy or radioiodine [8]. Although patients with PTC tend to have a good prognosis, a number of patients die from tumor proliferation and metastasis in a short time [9]. Therefore, it is crucial to elucidate the pathological mechanism of PTC, develop novel diagnostic strategy for the disease, and interfere with the thyroid neoplasm progress into malignant cancer.

Previous reports have demonstrated that long noncoding RNAs (lncRNAs) play roles in TC [9–11]. The lncRNA BRAF-activated long noncoding RNA (BANCR) can promote proliferation and inhibit apoptosis in TC cells [12]. In addition, changes in the expression of multiple microRNAs (miRNAs) may be a major mechanism in TC tumorigenesis and could be used in disease diagnosis [13, 14]. In PTC cells, several miRNAs (hsa-miR-220, hsa-miR-221, and hsa-miR-222) are significantly upregulated, while, several other miRNAs (hsa-let-7, hsa-miR-26, and hsa-miR-345) are significantly downregulated [15–18]. It is worth mentioning that the interaction between lncRNAs and miRNAs influences tumor development and progression [19]. In this study, we tried to identify potential diagnostic lncRNAs and miRNAs biomarkers in PTC. We performed the differential expression analysis of lncRNAs, miRNAs, and mRNAs. Then, we performed machine learning to find optimal diagnostic, differentially expressed lncRNAs and miRNAs for PTC. Subsequent analysis was based on these optimal diagnostic lncRNAs and miRNAs.

## 2. Materials and Methods

### 2.1. Data Retrieval

In the Cancer Genome Atlas (TCGA) database (http://sangerbox.com/TcgaDown), the clinical data of 506 PTC patients, lncRNA and mRNA data of 502 PTC patients, and miRNA data of 506 PTC patients were recorded. Transcriptome lncRNA, miRNA, and mRNA count data of PTC were obtained from primary solid tumor and normal solid tissue. Finally, the lncRNA and mRNA data (involving 502 cases and 58 controls) and miRNA data (involving 506 cases and 59 controls) were used for analysis.

### 2.2. Screening of Differentially Expressed lncRNAs, miRNAs, and mRNAs

The edgeR package in *R* was used for differential analysis of lncRNAs, miRNAs, and mRNAs. The false discovery rate (FDR) was calculated from multiple comparisons. Differentially expressed lncRNAs, miRNAs, and mRNAs were identified with the criteria of *p* < 0.05, abs (log2Fold Change) > 1, FDR < 0.05, abs (log2Fold Change) > 1, and FDR < 0.05, abs (log2Fold Change) > 1.5, respectively.

### 2.3. Identification of the Optimal Diagnostic lncRNAs and miRNAs

Firstly, importance value of each lncRNA/miRNA was ranked by the random forest algorithm. Then, the optimal number of features was found by subsequently adding one lncRNA/miRNA at a time in a top-down forward-wrapper approach. Optimal lncRNA/miRNA with diagnostic value for PTC were used to establish classification models including random forests (RF), decision tree (DT), and support vector machine (SVM). The “randomForests” package in *R* language, “rpart” package in *R* language, and “e1071” package in *R* language were used to establish the RF model, DT model, and SVM model, respectively. Diagnostic ability of classification prediction was evaluated by obtaining the area under a receiver operating characteristic (ROC) curve.

### 2.4. Network of lncRNA-mRNA-miRNA

Firstly, the Pearson correlation coefficient (*r*) of identified diagnostic lncRNAs and differentially expressed mRNA was estimated using the cor.test function in *R*. The standard of co-expression relation pair was |*r*| > 0.5 and *p* value ≤ 0.05. Secondly, the pairwise Pearson correlation coefficient (*r*) between identified diagnostic miRNAs and mRNAs was calculated. miRNA-target prediction tools (miRDB, TragetScan, miRanda, and PICTAR2) were used to predict target mRNAs of diagnostic miRNAs. Interaction network of diagnostic lncRNA-co-expressed mRNA and the selected diagnostic miRNA-target pairs with negative correlations were visualized by using Cytoscape.

### 2.5. Functional Analysis of mRNAs

In order to understand the biological function of mRNAs, we conducted Gene Ontology (GO) enrichment analysis through DAVID 6.8 database (https://david.ncifcrf.gov/). *p* < 0.01 was considered as statistically significant.

### 2.6. Validation and Diagnostic Analysis of lncRNAs and miRNAs on Public Gene Expression Dataset

The GEO dataset, GSE33630 [20, 21] (involving 49 cases and 45 controls), was used for expression validation and ROC analysis of lncRNAs. In addition, GSE104006 [22] (involving 57 cases and 11 controls) was used for expression validation and ROC analysis of miRNAs. The expression result of these lncRNAs/miRNAs was shown by box plots.

### 2.7. Ex Vivo Validation of lncRNAs, miRNAs, and mRNAs

The inclusion criteria of PTC patients were as follows: (1) patients were diagnosed with PTC according to pathological examination; (2) patients underwent surgery of PTC for the first time and received no adjuvant therapy before; and (3) patients had complete clinical data including medical history of present illness, family history, personal history, detailed physical examination data, and postoperative pathological data. The exclusion criteria of PTC patients were as follows: (1) patients had autoimmune thyroid disease, other malignant tumors, or viral infections; (2) patients with recurrence; (3) patients with incomplete clinical information; and (4) patients took psychotropic drugs for a long time. The tumor tissue and paracarcinoma tissue of 7 patients were collected. The tumor tissue and paracarcinoma tissue of these patients were collected for validation. All participating individuals provided informed consent with the approval of the ethics committee of the local hospital.

All tissue specimens (confirmed by the pathologist) were frozen continuously within 15 min after surgery and stored in the refrigerator at −80°C for use. Total RNA of the tissue and paracarcinoma tissue was extracted using the TRIzol reagent. 1 *µ*g RNA was applied to synthesize DNA by FastQuant cDNA first-strand synthesis kit (TIANGEN). Then real-time PCR was performed in the ABI 7300 real-time PCR system with SYBR® Green PCR Master Mix. GAPDH and ACTB were endogenous controls of mRNA. Hsa-U6 was endogenous controls of lncRNA. Relative lncRNA/miRNA/mRNA expression was analyzed fold change method.

### 2.8. Function Analysis of Identified lncRNAs

In order to further analyze the function of identified lncRNAs, PTC cell lines (PTC-UC3) were used for experiments on cell proliferation and invasion. To confer stable overexpression, a lentiviral vector was used for gene delivery [23, 24]. The lncRNA was PCR-amplified from a complementary deoxyribonucleic acid library. PCR products and plasmid were purified. When PTC-UC3 cells were 50% confluent, they were infected with overexpression vector by Lipofectamine 2000 (Invitrogen) for 24 h at 37°C. Then, the virus-containing medium was replaced with fresh complete medium. The PTC-UC3 cells were incubated for a further 48 h. Cell growth status was deciphered by MTT assays. The absorption of the solution was measured at 490 nm at various time points (0 h, 24 h, 48 h, and 73 h). Cell invasion assay was evaluated using Transwell inserts with Matrigel-coated membrane matrix. The cell migration rate was calculated.

## 3. Results

### 3.1. lncRNA, miRNA, and mRNA Expression Pattern

**Figure 1:**
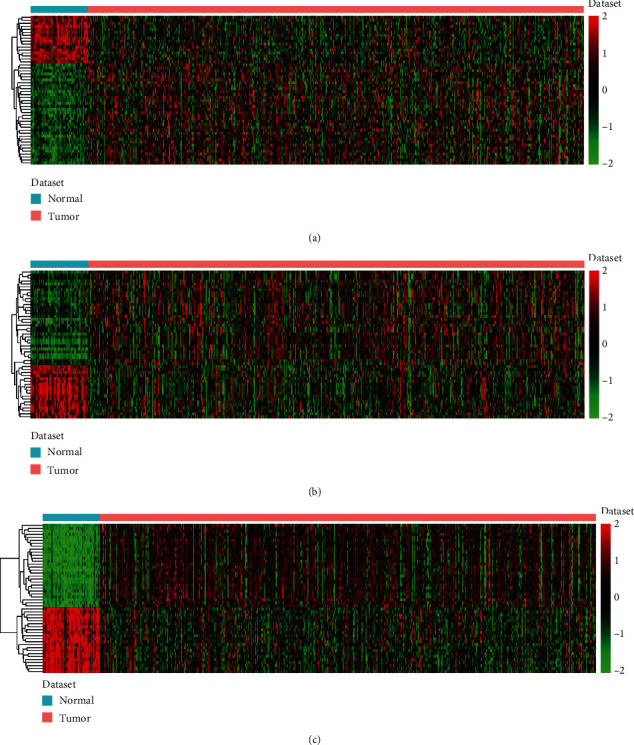
The heat maps of top 50 lncRNAs (a), top 50 miRNAs (b), and top 50 mRNAs (c) in PTC downloaded from the TCGA dataset. The diagram presents the result of a two-way hierarchical clustering of top 50 lncRNAs/miRNAs/mRNAs and samples. The differentially expressed lncRNA/miRNA/mRNA clustering tree is shown on the right.

Totally, 107 differentially expressed lncRNAs, 81 differentially expressed miRNAs, and 515 differentially expressed mRNAs were identified. The heat map of top 50 lncRNAs, top 50 miRNAs, and mRNAs is shown in Figures 1(a)–1(c), respectively.

### 3.2. Identification of Optimal Diagnostic lncRNAs and miRNAs

**Figure 2:**
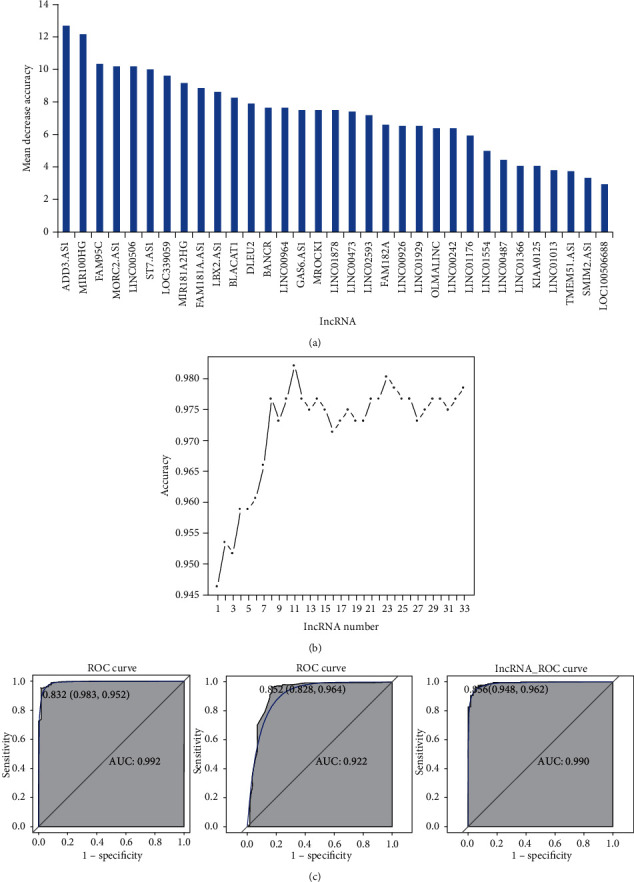
Identification of optimal diagnostic lncRNAs biomarkers for PTC based on RF, DT, and SVM classification models. (a) The ranking of all lncRNAs. lncRNAs were ranked according to the standardized drop in prediction accuracy. (b) The tendency chart of AUC along with the increase of lncRNAs. (c) ROC results of combinations of 11 optimal diagnostic lncRNAs biomarkers.

**Figure 3:**
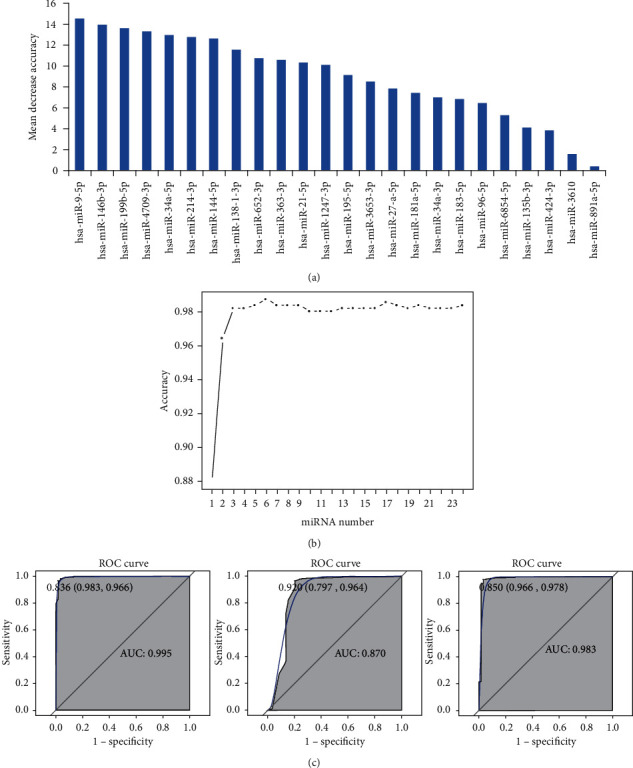
Identification of optimal diagnostic miRNAs biomarkers for PTC based on RF, DT, and SVM classification models. (a) The ranking of all miRNAs. miRNAs were ranked according to the standardized drop in prediction accuracy. (b) The tendency chart of AUC along with the increase of miRNAs. (c) ROC results of combinations of 6 optimal diagnostic lncRNAs biomarkers.

All differentially expressed lncRNAs were ranked according to the standardized drop in prediction accuracy (Figure 2(a)). 11 differentially expressed lncRNAs including ADD3-AS1 (downregulation), MIR100HG (downregulation), FAM95C (downregulation), MORC2-AS1 (downregulation), LINC00506 (downregulation), ST7-AS1 (downregulation), LOC339059 (downregulation), MIR181A2HG (upregulation), FAM181A-AS1 (downregulation), LBX2-AS1 (upregulation), and BLACAT1 (upregulation) were regarded as the optimal diagnostic biomarkers for PTC (Figure 2(b)). Above 11 optimal lncRNAs with diagnostic value for PTC were used to establish classification models. The AUC value in the RF, DT, and SVM models was 99.2%, 92.2%, and 99%, respectively (Figure 2(c)). All differentially expressed miRNAs were ranked according to the standardized drop in prediction accuracy (Figure 3(a)). Six differentially expressed miRNAs including hsa-miR-9-5p (downregulation), hsa-miR-146b-3p (upregulation), hsa-miR-199b-5p (downregulation), hsa-miR-4709-3p (upregulation), hsa-miR-34a-5p (upregulation), and hsa-miR-214-3p (downregulation) were considered as the optimal diagnostic biomarkers for PTC (Figure 3(b)). Above 6 optimal miRNAs with diagnostic value for PTC were used to establish classification models. The AUC value in the RF, DT, and SVM models was 99.5%, 87%, and 98.3%, respectively (Figure 3(c)).

### 3.3. Network of lncRNA-mRNA-miRNA

**Figure 4:**
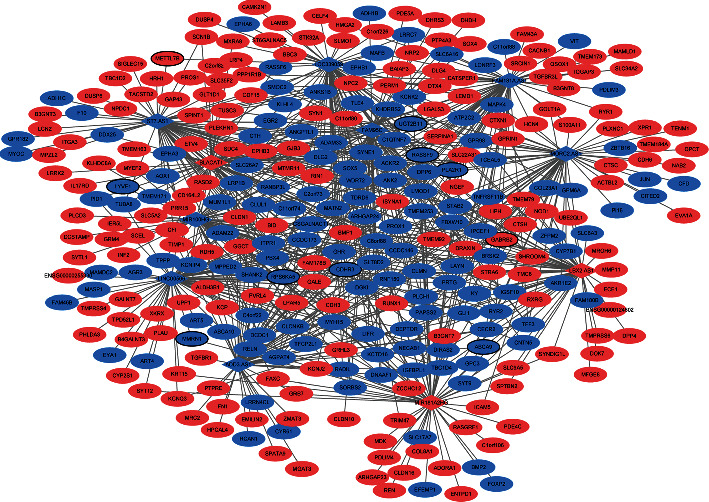
The interaction network of diagnostic lncRNA-co-expressed mRNA in PTC based on the cor.test function in *R*. Rhombus and ellipse represent diagnostic lncRNA and co-expressed mRNA, respectively; red and blue colors represent the upregulation and downregulation, respectively; the black border represents the top 10 upregulated and top 10 downregulated mRNA.

**Figure 5:**
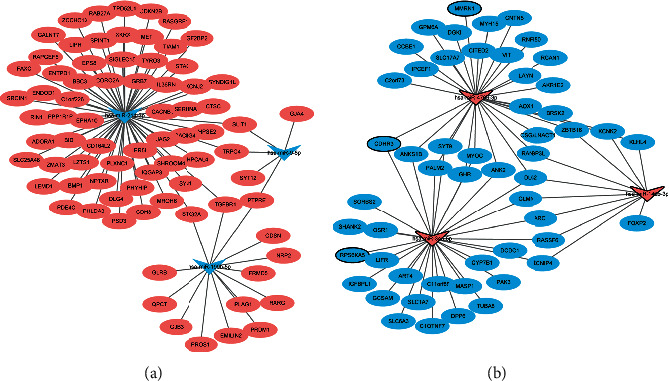
The network of diagnostic miRNA-target mRNAs with negative correlation in PTC based on miRNA-target prediction tools. The rectangle and ellipses represent the diagnostic miRNAs and target mRNAs, respectively; the pink and blue colors represent upregulation and downregulation, respectively; the black border represents the top 10 upregulated and top 10 downregulated mRNA.

**Figure 6:**
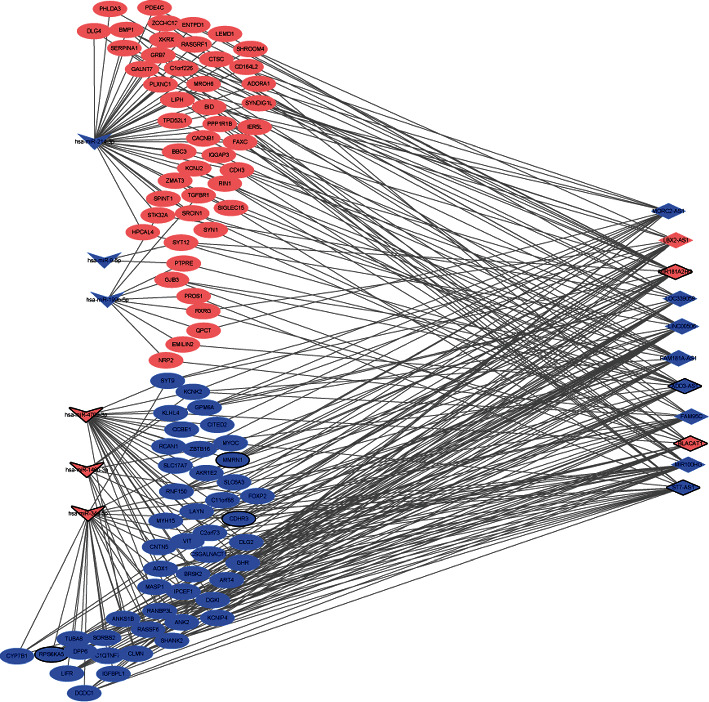
The ceRNA network of diagnostic lncRNA-common mRNA-diagnostic miRNA in PTC after taking the intersection of the interaction network of diagnostic lncRNA-co-expressed mRNA and diagnostic miRNA-mRNA pairs. The rhombus, rectangle, and ellipse represent the diagnostic lncRNAs, diagnostic miRNAs, and mRNAs, respectively; the pink and blue colors represent upregulation and downregulation, respectively; the black border represents the top 10 upregulated and top 10 downregulated lncRNA/miRNA/mRNA.

To investigate the interaction between diagnostic lncRNAs and mRNAs and diagnostic miRNAs and mRNAs, the ceRNA network of lncRNA-mRNA-miRNA was constructed. A total of 335 mRNAs were co-expressed with 11 diagnostic lncRNAs. The interaction network of diagnostic lncRNA-co-expressed mRNA is shown in Figure 4. In addition, 152 diagnostic miRNA-mRNA pairs (involving 6 miRNA and 130 mRNA) were identified in the targeted and negatively correlation analysis. The established regulatory network of diagnostic miRNA-targeted mRNA with negative correlation is shown in Figure 5. After taking the intersection, we obtained 95 common differentially expressed mRNAs between networks of diagnostic lncRNA-co-expressed mRNA and diagnostic miRNA-targeted mRNA. The ceRNA network of diagnostic lncRNA-common mRNA-diagnostic miRNA is shown in Figure 6. The ceRNA network contains 11 diagnostic lncRNAs, 6 diagnostic miRNAs, and 95 differentially expressed mRNAs. Several interaction pairs were identified, such as MIR181A2HG-FOXP2-hsa-miR-146b-3p, BLACAT1/ST7-AS1-RPS6KA5-hsa-miR-34a-5p, LBX2-AS1/MIR100HG-CDHR3-hsa-miR-34a-5p, ADD3-AS1-PTPRE-hsa-miR-9-5p, ADD3-AS1-TGFBR1-hsa-miR-214-3p, LINC00506-MMRN1-hsa-miR-4709-3p, and LOC339059-STK32A-hsa-miR-199b-5p.

### 3.4. Functional Enrichment of mRNAs

**Figure 7:**
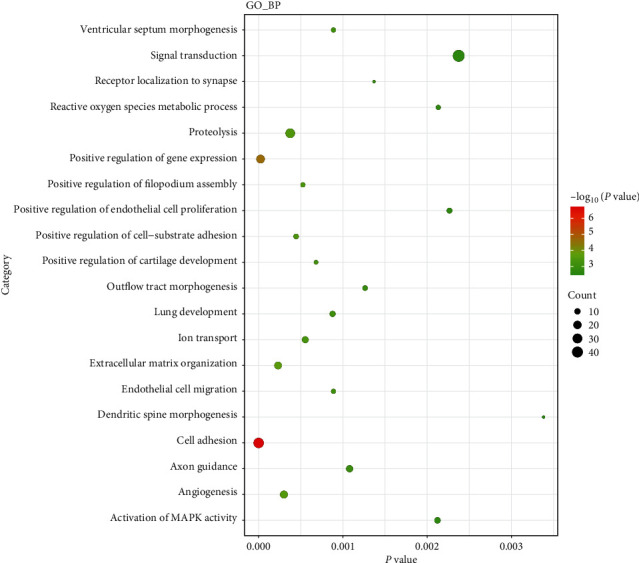
Top 20 significant enrichment BP terms of mRNAs in PTC based on GO enrichment analysis.

The GO analysis results demonstrated that these targeted differentially expressed mRNAs were significantly involved in some biological processes (BP), such as cell adhesion (involved MMRN1) and endothelial cell migration (involved TGFBR1) (Figure 7).

### 3.5. Validation and Diagnostic Analysis of lncRNAs and miRNAs on Public Gene Expression Dataset

**Figure 8:**
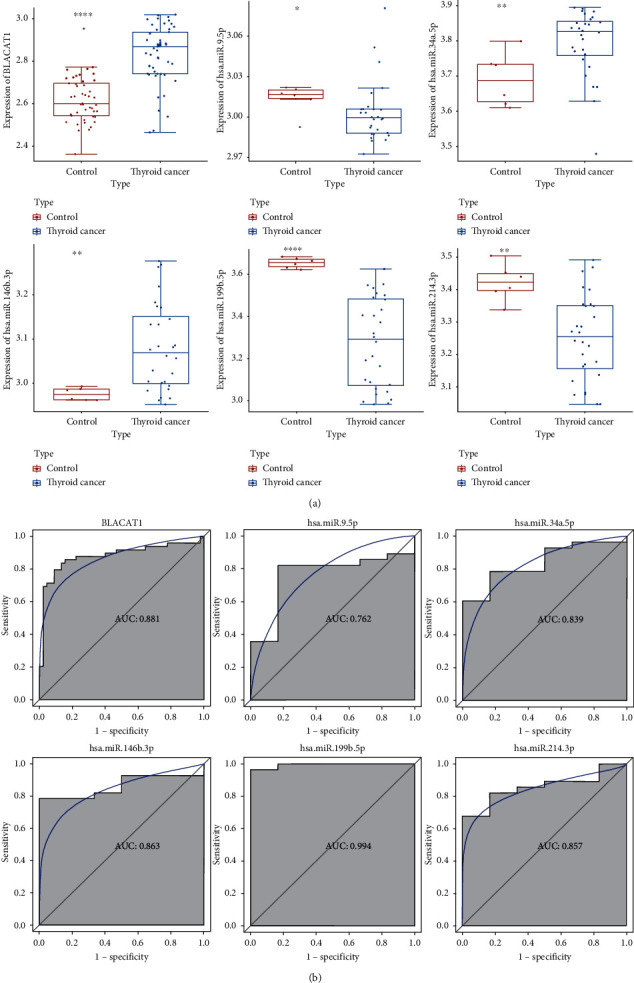
Electronic validation (a) and diagnostic analysis (b) of lncRNAs and miRNAs in PTC in the GSE33630 and GSE104006 datasets. ^*∗∗*^*p* < 0.01; ^*∗∗∗*^*p* < 0.001.

The GSE33630 was used for expression validation and ROC analysis of BLACAT1. Additionally, GSE104006 was used for expression validation and ROC analysis of hsa-miR-9-5p, hsa-miR-34a-5p, hsa-miR-146b-3p, hsa-miR-199b-5p, and hsa-miR-214-3p (Figures 8(a) and 8(b)). The expression of BLACAT1, hsa-miR-34a-5p, and hsa-miR-146b-3p was upregulated, while hsa-miR-9-5p, hsa-miR-199b-5p, and hsa-miR-214-3p were downregulated in PTC. Moreover, BLACAT1 (AUC = 0.881), hsa-miR-9-5p (AUC = 0.762), hsa-miR-34a-5p (AUC = 0.839), hsa-miR-146b-3p (AUC = 0.863), hsa-miR-199b-5p (AUC = 0.994), and hsa-miR-214-3p (AUC = 0.857) had a diagnostic value for PTC patients. The expression validation and diagnostic analysis were consistent with the informatics analysis.

### 3.6. Ex Vivo Validation of lncRNAs, miRNAs, and mRNAs

**Figure 9:**
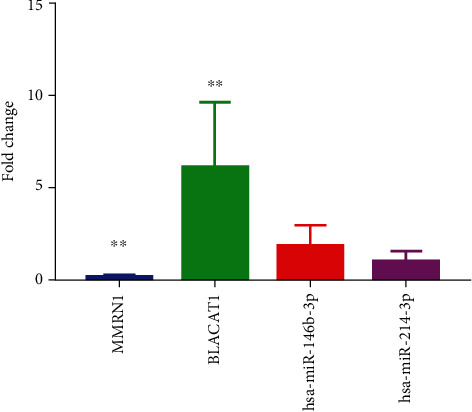
The ex vivo validation of BLACAT1, hsa-miR-146b-3p, hsa-miR-214-3p, and MMRN1 in 7 PTC patients. Fold change > 1 and fold change < 1 represent upregulation and downregulation, respectively. ^*∗∗*^*p* < 0.01.

The ex vivo experiment was used to further validate the expression of identified lncRNA (BLACAT1), miRNAs (hsa-miR-146b-3p and hsa-miR-214-3p), and mRNA (MMRN1) in 7 PTC patients. Clinical information of 7 patients is presented in Table 1. The expression of BLACAT1 and hsa-miR-146b-3p was significantly upregulated, while the expression of hsa-miR-214-3p and MMRN1 was downregulated in PTC without significance (Figure 9). The expression trend of these molecules was in line with the bioinformatics analysis.

### 3.7. Function Analysis of ADD3-AS1

**Figure 10:**
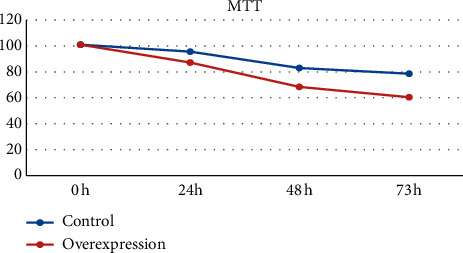
Cell proliferation of PTC-UC3 after ADD3-AS1 overexpression was detected by MTT assay.

**Figure 11:**
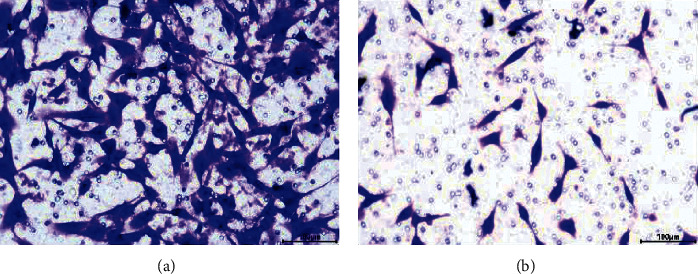
Effects of ADD3-AS1 overexpression on PTC-UC3 cells invasion. Scale: 100 *μ*m. (a) Control. (b) Overexpression.

According to the bioinformatics analysis and related literature report, we selected one of lncRNAs ADD3-AS1 for function analysis. The MTT assay showed that overexpression of ADD3-AS1 significantly inhibited cell proliferation at 24 h, 48 h, and 73 h (Figure 10). The cell invasion result indicated that overexpression of ADD3-AS1 decreased the PTC-UC3 cells' invasion capacity compared with the normal control (Figure 11). These results demonstrated that overexpression of ADD3-AS1 inhibited the proliferation and invasion of PTC-UC3 cells.

## 4. Discussion

In our study, we found that identified lncRNAs/miRNAs/mRNA were differentially expressed between normal and cancerous tissues. In addition, 11 lncRNAs (MIR181A2HG, BLACAT1, ST7-AS1, LBX2-AS1, MIR100HG, ADD3-AS1, LINC00506, LOC339059, MORC2-AS1, FAM95 C, and FAM181A-AS1) and 6 miRNAs (hsa-miR-146b-3p, hsa-miR-34a-5p, hsa-miR-9-5p, hsa-miR-214-3p, hsa-miR-4709-3p, and hsa-miR-199b-5p) were considered as the optimal diagnostic biomarkers for PTC.

Upregulation of MIR181A2HG is significantly associated with overall survivability of TC patients [25]. Hsa-miR-146b-3p is increased in TC and PTC [26, 27]. We found that MIR181A2HG and hsa-miR-146b-3p also increased in PTC. Significantly, MIR181A2HG and hsa-miR-146b-3p had a diagnostic value for PTC. In addition, downregulated forkhead box P2 (FOXP2) was co-expressed with MIR181A2HG and targeted by hsa-miR-146b-3p. FOXP2 is downregulated in tumor tissues of hepatocellular carcinoma; its downregulation remarkably promotes the invasiveness of hepatocellular carcinoma [28]. The downregulation of FOXP2 is found in PTC [29]. Our result indicated that interaction pairs of MIR181A2HG-FOXP2-hsa-miR-146b-3p may be associated with PTC.

The expression of BLACAT1 is increased in some cancers, such as medulloblastoma, colon adenocarcinoma, pancreatic adenocarcinoma, gastric cancer, and TC [30–35]. Moreover, BLACAT1 has a prognostic value for patients with PTC [36]. The downregulation of ST7-AS1 has been found in cervical cancer, PTC, and TC [35, 37]. The expression levels of hsa-miR-34a-5p are upregulated in tumor samples of PTC [38]. In addition, hsa-miR-34a-5p is a cancer biomarker of TC [39]. Similarly, the upregulation of BLACAT1 and downregulation of ST7-AS1 were found in this study. Furthermore, BLACAT1, ST7-AS1, and hsa-miR-34a-5p had a remarkably diagnostic value for PTC. Additionally, we found that downregulated ribosomal protein S6 kinase A5 (RPS6KA5) was co-expressed with both BLACAT1 and ST7-AS1. Furthermore, RPS6KA5 was one of the targets of hsa-miR-34a-5p. It has been demonstrated that RPS6KA5 is downregulated in tumor tissues of PTC [29]. This suggested that BLACAT1/ST7-AS1-RPS6KA5-hsa-miR-34a-5p played a crucial regulatory role in PTC.

LBX2-AS1 is upregulated in PTC and TC [37]. MIR100HG is downregulated in pediatric medulloblastoma [31]. In gastric cancer, downregulation of MIR100HG inhibits cell proliferation and migration [40]. In triple-negative breast cancer, reduced expression of MIR100HG leads to a reduction in cell proliferation [41]. It is reported that MIR100HG is significantly associated with overall survival of PTC patients [42]. We found that LBX2-AS1 and MIR100HG were, respectively, upregulated and downregulated in PTC tumor. In addition, cadherin-related family member 3 (CDHR3) was co-expressed with LBX2-AS1 and MIR100HG. Moreover, CDHR3 was targeted by hsa-miR-34a-5p. It is indicated that the epigenetic regulation between LBX2-AS1, MIR100HG, hsa-miR-34a-5p, and CDHR3 may provide a novel field in understanding the pathological mechanism of PTC.

ADD3-AS1 is downregulated in PTC and TC [35, 43]. In PTC, hsa-miR-9-5p is downregulated and plays an important role by targeting B-Raf proto-oncogene and serine/threonine kinase (BRAF) [44]. Hsa-miR-9-5p is downregulated in anaplastic TC, suggesting its role as a potential tumor suppressor in anaplastic TC [45]. We found that ADD3-AS1 and hsa-miR-9-5p were downregulated in PTC, which agrees with previous studies. Moreover, upregulated protein tyrosine phosphatase receptor type *E* (PTPRE) was co-expressed with ADD3-AS1 and targeted by hsa-miR-9-5p. Compared with benign PTC, PTPRE is upregulated in malignant PTC [46]. In the function analysis, we found that overexpression of ADD3-AS1 inhibited the proliferation and invasion of PTC-UC3 cells, which suggested the important role of ADD3-AS1 in the tumorigenesis of PTC. In addition, our study implied that the interaction pairs of ADD3-AS1-PTPRE-hsa-miR-9-5p play a crucial role in PTC.

It is revealed that hsa-miR-214-3p is decreased in hepatocellular carcinoma and non-small-cell lung cancer [47, 48]. hsa-miR-214-3p is involved in PTC [37]. We found that the expression of hsa-miR-214-3p decreased in PTC. In addition, upregulated transforming growth factor beta receptor 1 (TGFBR1) was co-expressed with ADD3-AS1 and one of the targets of hsa-miR-214-3p. The expression of TGFBR1 is upregulated in PTC and TC [29, 49]. In PTC, SLC35F2 expedites the proliferation and migration of tumor cells by targeting TGFBR1 [50]. Our finding indicated that the ceRNA network of ADD3-AS1-TGFBR1-hsa-miR-214-3p could be involved in PTC proliferation and migration.

LINC00506 is downregulated in TC [35]. hsa-miR-4709-3p is a dysregulated miRNA in different brain regions of patients with autism spectrum disorders [51]. In this study, we found that LINC00506 was decreased, while hsa-miR-4709-3p was upregulated in PTC. Interestingly, both LINC00506 and hsa-miR-4709-3p had a diagnostic value for PTC patients. In addition, downregulated multimerin 1 (MMRN1) was co-expressed with LINC00506 and targeted by hsa-miR-4709-3p. MMRN1 is downregulated in chronic lymphocytic leukemia and PTC [51, 52]. Our result indicated that the interaction pairs of LINC00506-MMRN1-hsa-miR-4709-3p may be associated with PTC.

Up to now, no association between LOC339059 and cancer has been found in previous reports. In PTC, downregulated hsa-miR-199b-5p can predict tumor aggressiveness and is a diagnostic blood marker of PTC [53, 54]. In follicular TC, hsa-miR-199b-5p is downregulated, indicating its potential to promote malignant transformation and an important diagnostic tool [55]. In medullary TC, downregulated hsa-miR-199b-5p may function as a carcinogen or tumor suppressor [56]. Herein, we found that LOC339059 and hsa-miR-199b-5p were downregulated and had a remarkably diagnostic value for PTC patients. In addition, upregulated serine/threonine kinase 32A (STK32 A) was co-expressed with LOC339059 and targeted by hsa-miR-199b-5p. The expression of STK32 A has been found in familial nonmedullary TC and PTC [43, 57]. This suggested that LOC339059-STK32A-hsa-miR-199b-5p played a crucial role in PTC.

Besides above lncRNAs, the remaining 3 downregulated diagnostic lncRNAs (MORC2-AS1, FAM95C, and FAM181A-AS1) may play roles in PTC. MORC2-AS1, FAM95C, and FAM181A-AS1 are downregulated in TC [35, 37, 43]. In addition, we found that MMRN1 and TGFBR1 were, respectively, involved in cell adhesion and endothelial cell migration according to the GO functional analysis. Some neural cell adhesion molecules are expressed normally in thyroid follicular cells. It is found that various pathways, such as cell adhesion molecules, were increased in PTC [58]. The endothelial cell migration is a crucial prerequisite for tumor angiogenesis. It has been demonstrated that endothelial cell migration is involved in TC [59–61].

## 5. Conclusions

We got numbers of differentially expressed lncRNAs, miRNAs, and mRNAs, which were differentially expressed between normal and cancerous tissues. According to the machine learning, 11 lncRNAs and 6 miRNAs were regarded as the optimal diagnostic biomarkers for PTC. The epigenetic modifications of MIR181A2HG-FOXP2-hsa-miR-146b-3p, BLACAT1/ST7-AS1-RPS6KA5-hsa-miR-34a-5p, LBX2-AS1/MIR100HG-CDHR3-hsa-miR-34a-5p, ADD3-AS1-PTPRE-hsa-miR-9-5p, ADD3-AS1-TGFBR1-hsa-miR-214-3p, LINC00506-MMRN1-hsa-miR-4709-3p, and LOC339059-STK32A-hsa-miR-199b-5p may be involved in tumorigenesis of PTC. However, there are limitations to our study. Firstly, the sample of ex vivo validation is small, and a larger number of PTC samples are further needed. Secondly, the deeper mechanism study of the PTC is also needed in animal models. Thirdly, the differences between PTC subtypes, such as anaplastic TC with aggressiveness and poor outcome, should be analyzed in the further study.

## Figures and Tables

**Table 1:** Clinical information of 7 PTC patients.

Number	Age	TNM classification (AJCC 2002 edition)	Tumor infiltration	TOTT3 (nmol/L)	TOTT4 (nmol/L)	TSH (4.19 mIU/L)	Complications	Family history
1	50	T1aN0M0	No	1.53	118.52	2.64	No	No
2	53	mT1aN0M0	No	1.13	138.28	2.88	No	No
3	47	mT1aN0M0	No	1.52	102.52	1.19	Hypertension	No
4	40	T3bN1aM0	No	1.29	94.42	4.98	No	No
5	49	T1bN0M0	No	1.75	104.48	0.63	Hypertension	No
6	54	T3bN1bM0	Yes	1.53	126.58	1.40	Hypertension	No
7	52	T3bN1aM0	No	1.38	118.48	4.19	No	No

AJCC: American Joint Committee on Cancer; TOTT3: total triiodothyronitrogenic acid; TOTT4: total thyroxine; and TSH: thyroid-stimulating hormone.

## Data Availability

All data used to support the study are included within the article.
